# Accuracy and reproducibility of simplified QSPECT dosimetry for personalized ^177^Lu-octreotate PRRT

**DOI:** 10.1186/s40658-018-0224-9

**Published:** 2018-10-15

**Authors:** Michela Del Prete, Frédéric Arsenault, Nassim Saighi, Wei Zhao, François-Alexandre Buteau, Anna Celler, Jean-Mathieu Beauregard

**Affiliations:** 1 Department of Radiology and Nuclear Medicine and Cancer Research Center, Université Laval, Quebec City, Canada; 2 Department of Medical Imaging and Oncology Branch of CHU de Québec Research Center, CHU de Québec – Université Laval, 11 côte du Palais, Quebec City, QC G1R 2J6 Canada; 3 Medical Imaging Research Group, University of British Columbia, Vancouver, Canada; 4 Department of Physics and Astronomy, University of British Columbia, Vancouver, Canada

**Keywords:** Dosimetry, Neuroendocrine tumors, Peptide receptor radionuclide therapy, Personalized, Quantitative SPECT

## Abstract

**Background:**

Routine dosimetry is essential for personalized ^177^Lu-octreotate peptide receptor radionuclide therapy (PRRT) of neuroendocrine tumors (NETs), but practical and robust dosimetry methods are needed for wide clinical adoption. The aim of this study was to assess the accuracy and inter-observer reproducibility of simplified dosimetry protocols based on quantitative single-photon emission computed tomography (QSPECT) with a limited number of scanning time points. We also updated our personalized injected activity (IA) prescription scheme.

**Methods:**

Seventy-nine NET patients receiving ^177^Lu-octreotate therapy (with a total of 279 therapy cycles) were included in our study. Three-time-point (3TP; days 0, 1, and 3) QSPECT scanning was performed following each therapy administration. Dosimetry was obtained using small volumes of interest activity concentration sampling for the kidney, the bone marrow and the tumor having the most intense uptake. Accuracy of the simplified dosimetry based on two-time-point (2TP; days 1 and 3, monoexponential fit) or a single-time-point (1TP_D3_; day 3) scanning was assessed, as well as that of hybrid methods based on 2TP for the first cycle and 1TP (day 1 or 3; 2TP/1TP_D1_ and 2TP/1TP_D3_, respectively) or no imaging at all (based on IA only; 2TP/no imaging (NI)) for the subsequent induction cycles. The inter-observer agreement was evaluated for the 3TP, 2TP, and hybrid 2TP/1TP_D3_ methods using a subset of 60 induction cycles (15 patients). The estimated glomerular filtration rate (eGFR), body size descriptors (weight, body surface area (BSA), lean body weight (LBW)), and products of both were assessed for their ability to predict IA per renal absorbed dose at the first cycle.

**Results:**

The 2TP dosimetry estimates correlated highly with those from the 3TP data for all tissues (Spearman *r* > 0.99, *P* < 0.0001) with small relative errors between the methods, particularly for the kidney and the tumor, with median relative errors not exceeding 2% and interdecile ranges spanning over less than 6% and 4%, respectively, for the per-cycle and cumulative estimates. For the bone marrow, the errors were slightly greater (median errors < 6%, interdecile ranges < 14%). Overall, the strength of correlations of the absorbed dose estimates from the simplified methods with those from the 3TP scans tended to progressively decrease, and the relative errors to increase, in the following order: 2TP, 2TP/1TP_D3_, 1TP_D3_, 2TP/1TP_D1_, and 2TP/NI. For the tumor, the 2TP/NI scenario was highly inaccurate due to the interference of the therapeutic response. There was an excellent inter-observer agreement between the three observers, in particular for the renal absorbed dose estimated using the 3TP and 2TP methods, with mean errors lesser than 1% and standard deviations of 5% or lower. The eGFR · LBW and eGFR · BSA products best predicted the ratio of IA to the renal dose (GBq/Gy) for the first cycle (Spearman *r* = 0.41 and 0.39, respectively; *P* < 0.001). For the first cycle, the personalized IA proportional to eGFR · LBW or eGFR · BSA decreased the range of delivered renal absorbed dose between patients as compared with the fixed IA. For the subsequent cycles, the optimal personalized IA could be determined based on the prior cycle renal GBq/Gy with an error of less than 21% in 90% of patients.

**Conclusions:**

A simplified dosimetry protocol based on two-time-point QSPECT scanning on days 1 and 3 post-PRRT provides reproducible and more accurate dose estimates than the techniques relying on a single time point for non-initial or all cycles and results in limited patient inconvenience as compared to protocols involving scanning at later time points. Renal absorbed dose over the 4-cycle induction PRRT course can be standardized by personalizing IA based on the product of eGFR with LBW or BSA for the first cycle and on prior renal dosimetry for the subsequent cycles.

## Background

For patients with metastatic neuroendocrine tumors (NETs), peptide receptor radionuclide therapy (PRRT) with ^177^Lu-octreotate is an effective palliative treatment that rarely causes serious toxicity [1, 2]. PRRT has been mostly administered as a 4-cycle induction course using a fixed injected activity (IA) of not more than 7.4 GBq per cycle, in order to not exceed cumulative absorbed doses of 23 Gy to the kidney and 2 Gy to the bone marrow (BM) in the majority of patients [1–4]. However, it is well known that for these critical organs, and in particular for the kidney which is the dose-limiting organ for most patients, the absorbed dose per IA is highly variable and usually lower than 23 Gy per 4 cycles, resulting in most patients being undertreated with such an empiric PRRT regime [5, 6]. We and others have proposed personalized PRRT (P-PRRT) protocols in which the number of fixed IA cycles or the IA per cycle are modulated to deliver a safe prescribed renal absorbed dose, with the aim to maximize tumor irradiation while keeping the toxicity low [4, 6]. Such P-PRRT protocols require careful dosimetry monitoring, which is often perceived as a complex and resource-consuming process, therefore constituting a barrier for wide clinical adoption. As a result, the clinical practice of “one-size-fits-all” PRRT prevails, at the potential cost of delivering a suboptimal treatment to most patients.

We have been routinely performing post-PRRT dosimetry using quantitative single-photon emission computed tomography (QSPECT) combined with the small-sphere volume of interest (VOI) activity concentration sampling [5, 6]. Aiming to simplify the dosimetry process and to reduce the clinical burden thereof, we examined the impact of reducing the number of QSPECT sessions on the accuracy and the inter-observer reproducibility of the resulting dose estimates. In parallel, based on a large dataset from our growing cohort of patients treated with PRRT, we updated our personalized IA determination scheme.

## Methods

### Patients and PRRT cycles

From November 2012 to December 2017, 81 patients with progressive metastatic and/or symptomatic NET were treated with PRRT at CHU de Québec—Université Laval. Two patients who underwent only 1 cycle were each excluded because of their incomplete dosimetric data, and therefore, only data from 79 patients was analyzed. This includes 23 patients who received only empiric PRRT (i.e., fixed IA of approximately 8 GBq, occasionally reduced) until March 2016, for whom the requirement for consent was waived due to the retrospective nature of the analysis. All other patients were enrolled in our P-PRRT trial (NCT02754297) and gave informed consent to participate (protocol described in [6]). Patient characteristics are reported in Table 1.

**Table 1:** Patient characteristics

	All patients (*n* = 79)
Gender, *n* (%)	
Female	36 (45.6)
Male	43 (54.4)
Age at first cycle, median (range)	60.7 (26.1–82.3)
Site of primary tumor, *n* (%)	
Small intestine	30 (38.0)
Pancreas	26 (32.9)
Adrenal gland^a^	6 (7.6)
Lung	6 (7.6)
Colon	2 (2.5)
Stomach	1 (1.3)
Esthesioneuroblastoma	1 (1.3)
Unknown	7 (8.9)
Metastases, *n* (%)	
Liver	66 (83.5)
Lymph nodes	51 (64.6)
Bone	29 (36.7)
Lung	9 (11.4)
Other^b^	25 (31.6)
Body size descriptors, mean ± SD (range)	
Weight (Kg)	72.1 ± 16.6 (42.6–121.0)
Lean body weight (Kg)	52.4 ± 9.9 (35.4–81.2)
Body surface area (m^2^)	1.8 ± 80.2 (1.4–2.5)
eGFR (ml/min/1.73 m^2^), mean ± SD (range)	86.3 ± 22.2 (42.0–154.1)
Number of cycles, *n* (%)	
1	8 (10.1)
2	6 (7.6)
3	16 (20.3)
4	38 (48.1)
5	3 (3.8)
6	6 (7.6)
7	1 (1.3)
8	1 (1.3)
Type of cycles, *n* (%)	
Empiric only	23 (29.1)
Personalized only	45 (57.0)
Mixed	11 (13.9)

*eGFR* estimated glomerular filtration rate, *PRRT* peptide receptor radionuclide therapy

^a^Three patients with pheochromocytoma and three patients with paraganglioma

^b^Peritoneum, ovary, subcutaneous, pleura, meninges

Two hundred and eighty-four therapy cycles were administered during the study period. Five cycles in five patients were excluded from the analysis because of dosimetry protocol deviation or missing data. Among the 279 therapy cycles analyzed, 142 were empiric (median IA = 7.6 GBq; range, 3.8–9.1 GBq) and 137 were personalized (median IA = 9.0 GBq; range, 0.7–32.4 GBq). Anti-nausea premedication (ondansetron and dexamethasone) and a nephroprotective amino acid solution (lysine and arginine) were administered [1]. We administer a 4-cycle induction course for which the prescribed cumulative renal dose is 23 Gy (5 Gy at the first cycle; two-monthly intervals) and, in responders only, we offer consolidation, maintenance, and/or salvage cycles (prescribed renal dose of 6 Gy each; personalized intervals). As previously described, prescribed renal absorbed radiation doses were reduced in patients with renal or bone marrow impairment [6].

### Reference dosimetry method

**Fig. 1:**
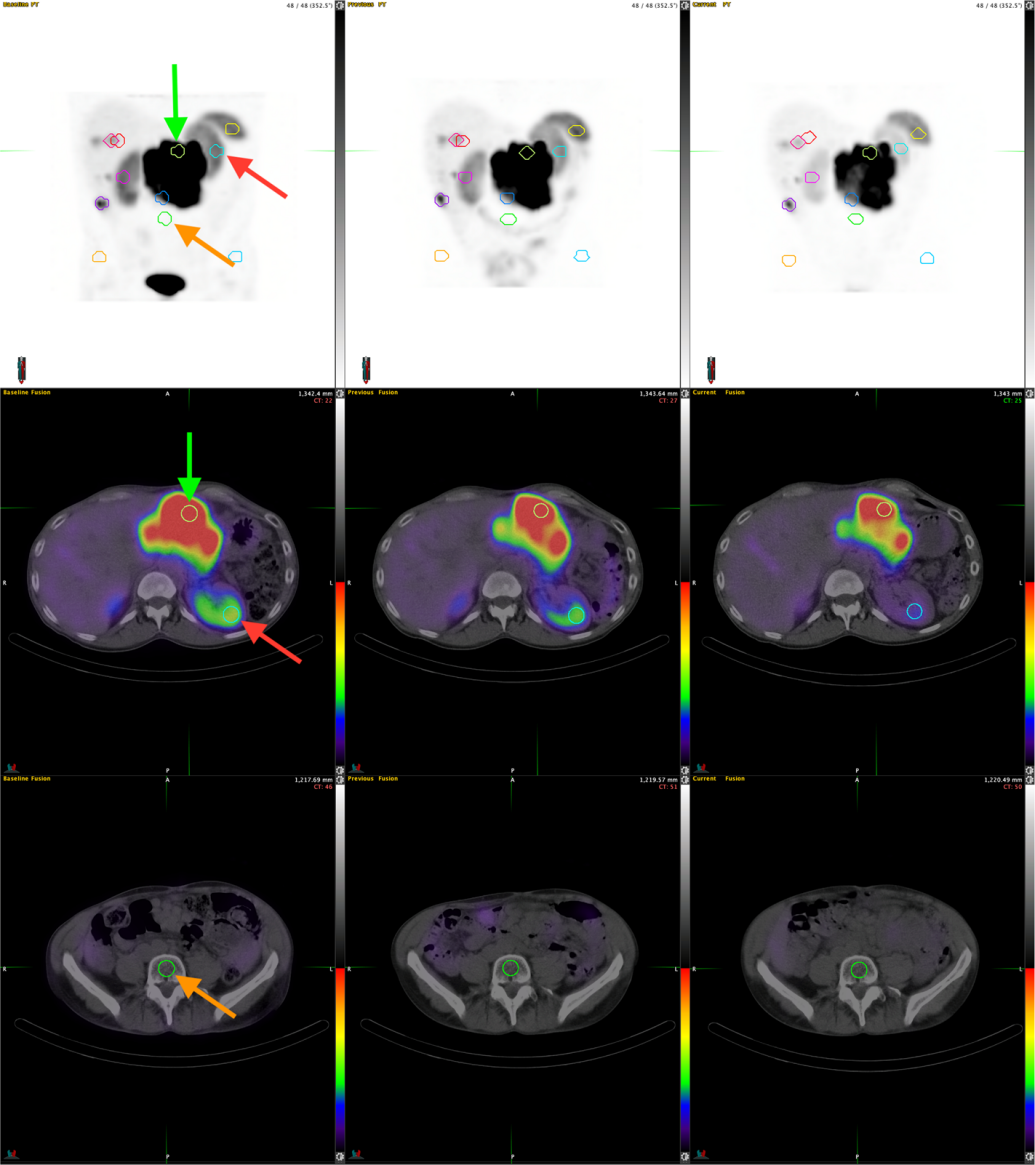
Post-treatment serial QSPECT/CT was performed at (from left to right) 5, 24, and 70 h after a 22.0 GBq ^177^Lu-octreotate administration in a 55-year-old male with metastatic NET of unknown origin. Small volumes of interest (2-cm diameter) were placed over tissues of interest. Left kidney (red arrows), L5 bone marrow cavity (orange arrows), and dominant tumor (green arrows) VOIs are pointed on anterior maximum intensity projections (top row) and selected transaxial fusion slices (mid and bottom rows). QSPECT images are normalized using an upper SUV threshold of 7. During this consolidation cycle, the personalized injected activity allowed the delivery of 6.1 Gy (6.0 Gy prescribed) to the kidney

At each cycle, after therapeutic administration of ^177^Lu-octreotate, QSPECT/computed tomography (QSPECT/CT) scans were performed at approximately 4 h (day 0), 24 h (day 1), and 72 h (day 3) using a Symbia T6 camera (Siemens Healthcare, Erlangen, Germany) (Fig. 1) [6, 7]. Following the same data processing as described in [7], the dead-time corrected reconstructed images were converted into the positron emission tomography (PET) DICOM format, which includes a “rescale slope” parameter that converts count data into Bq/mL and also enables display of QSPECT images in standardized uptake values normalized for body weight (standardized uptake value; SUV).

These three imaging time points were initially selected for the following practical reasons: (1) the day 0 scan does not incur any additional hospital visit for the patient and allows capturing early kinetics of the radiopharmaceutical and (2) performing scans beyond day 3 would not be easy for logistical reasons (PRRT being administered on Tuesday, day 4 or 5 would fall on the weekend) and would inconvenience patients (in particular those out-of-city patients who would need to prolong their stay).

As in our clinical practice, we routinely performed dosimetry based on the data acquired at these three time points (3TP), this approach constituted the reference method for the present analysis. In brief, at each time point, we sampled the activity concentration in tissues of interest (Fig. 1), including both kidneys (areas of representative parenchymal uptake), the BM (L4 and L5 vertebral bodies, or elsewhere when the latter were obviously affected by metastases), and the tumor having the most intense uptake (Tumor_max_), using 2-cm (4.2 cm^3^) spherical VOIs, as previously described [5, 6]. This was performed using either Hybrid Viewer (Hermes Medical Solutions, Stockholm, Sweden) or MIM Encore (MIM Software Inc., Cleveland, OH, USA) software. As previously described in [6], we also computed the total body retention for the purpose of computing the cross-dose component of the BM absorbed dose (BM_cross_), which we added to the self-dose component (BM_self_) to estimate the total BM absorbed dose (BM_total_).

**Fig. 2:**
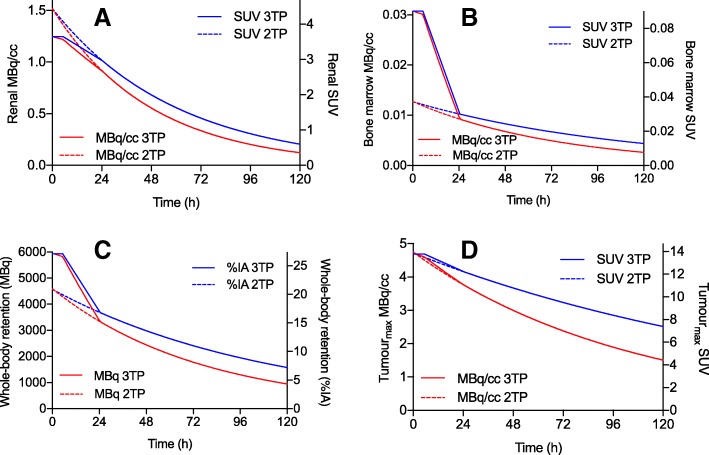
Time-activity curves (TACs) of the renal (**a**), tumor (**b**), and bone marrow (**c**) activity concentrations and of the whole-body retention (**d**) over time for the patient case illustrated in Fig. 1. TACs in MBq/cc or MBq (red) and SUV or percentage of injected activity (%IA) (blue) are illustrated for the three-time-point (3TP; solid lines) and two-time-point (2TP; dashed lines) method

Based on these 3TP data, trapezoidal-monoexponential (3TP_TM_) time-activity curves (TACs) were drawn using the following procedure (Fig. 2). For each organ/tumor, a constant mean SUV was assumed from the time of ^177^Lu-octreotate injection until the time of the day 0 scan (approximately 4 h). This was followed by a linear (trapezoid) fit to the SUV corresponding to the day 0 and the day 1 scans. Then, a monoexponential curve was fit using the day 1 and day 3 data, resulting in an effective decay model being used from day 1 onwards (trapezoidal-monoexponential; 3TP_TM_; Fig. 2). However, in cases when the day 3 SUV was higher than that corresponding to day 1, we assumed a linear SUV variation between days 1 and 3, followed by the physical decay of activity (i.e., *λ*_biol_ = 0, *λ*_eff_ = *λ*_phys_) from day 3 to infinity (trapezoidal-constant; 3TP_TC_).

Then, the area under each TAC curve was integrated and multiplied by the appropriate activity concentration dose factors (ACDF). The values of these factors have been derived from OLINDA/EXM software data (Vanderbilt University, Nashville, TN, USA), as previously described [6]: 84 mGy · g/MBq/h for Tumor_max_ and 87 mGy · g/MBq/h for kidneys and BM_self_. For BM_cross_, we integrated the total body activity over time and multiplied it by a dose factor of 1.09 × 10^−4^ mGy/MBq/h for males or 1.29 × 10^−4^ mGy/MBq/h for females, i.e., to account for their different gamma fraction of energy deposition from the whole body to the BM [6].

### Simplified dosimetry methods

From our experience and as suggested by others, the day 0 data, although it captures the rapid kinetics of the radiopharmaceutical (which includes competing accumulation and rapid washout), contributes little to the area under the TAC, which is mostly determined by the slow washout kinetics and tends to follow a monoexponential decay beyond 24 h [8]. Accordingly, we eliminated the day 0 data from all simplified dosimetry approaches. A total of five methods were investigated, as detailed below.

*2TP*: Two-time-point (2TP) dosimetry estimates were obtained using VOI data from day 1 to day 3 scans. From time of administration to infinity, monoexponential (2TP_M_) effective decay was applied, except in cases of biological accumulation of activity, i.e., when the SUV of the tissue increased between day 1 and day 3. In such cases, we assumed a SUV equal to that of day 3 SUV (*λ*_biol_ = 0), from time of treatment administration to infinity and thus applied only physical decay (*λ*_eff_ = *λ*_phys_; 2TP_C_). The 2TP method is the combination of 2TP_M_ and 2TP_C_.

*1TP*_*D3*_: As proposed by Hänscheid et al., we estimated doses using a single-time-point method based on the day 3 data (1TP_D3_) [8]. In this method, the activity concentration (MBq/cc) was multiplied by the time at which the day 3 scan was performed (h) and by 0.25 Gy · g/MBq/h (based on Eq. 8 in [8]). To compute BM_cross_, the total whole-body activity (MBq) was multiplied by imaging time (h) and by 3.2 × 10^−7^ Gy/MBq/h for males or 3.7 × 10^−7^ Gy/MBq/h for females, i.e., the gamma fraction of energy deposition from the whole body to the BM, multiplied by 0.25 Gy · g/MBq/h (from [8], as above), divided by 87 mGy · g/MBq/h (ACDF of the BM and kidney).

*2TP/1TP*_*D1*_: We evaluated a hybrid dosimetry protocol based on the 2TP method for the first cycle, as described above, and employed a single scan on day 1 for the subsequent induction cycles. In this scenario, the absorbed dose to a given tissue during the second and the subsequent induction cycles was obtained by applying the monoexponential curve corresponding to the effective decay, as determined for this tissue during the first cycle, to the activity concentration observed on day 1 of the subsequent cycle.

*2TP/1TP*_*D3*_: This is the same as 2TP/1TP_D1_, but the single scan on subsequent cycles was that performed on day 3.

*2TP/NI*: Similar to the two previous methods, this method was also based on 2TP scanning for the first cycle, but no imaging (NI) was performed for the subsequent cycles. For the latter, the absorbed dose per IA during the subsequent cycles was simply assumed to be equal to that delivered during the first cycle.

Cumulative renal, BM_total_, and Tumor_max_ absorbed doses were compiled for all patients who received three or four induction cycles (*n* = 65). Per-cycle and cumulative doses resulting from each of the simplified dosimetry methods were compared with those obtained using the reference (3TP) method, and relative errors were calculated.

### Inter-observer variability

For 60 induction cycles in 15 patients, the dosimetry analysis was performed independently by three observers having different backgrounds and purposely varied levels of experience in internal dosimetry. Observer 1 (M.D.P.), a certified endocrinologist, current PRRT Fellow and Ph.D. student, performed 258 of the 279 primary analyses described in this paper and, as such, accumulated the most experience with this dosimetry procedure. Observer 2 (F.A.) was a certified nuclear medicine physician and current Nuclear Oncology fellow who performed 21 primary analyses. Observer 3 (N.S.) was an M.D. student who was new to both nuclear medicine and dosimetry and who received only a short training. Relative errors of per-cycle and cumulative absorbed doses between each pair of observers were computed for the reference method (3TP) and the two most accurate simplified methods.

### Personalized ^177^Lu-octreotate activity prescription

We previously derived a model based on the body surface area (BSA) and the estimated glomerular filtration rate (eGFR; according to the CKD-EPI Creatinine Equation [9]) to determine the personalized ^177^Lu-octreotate activity to be administered at the first cycle [6]. Using data from our entire cohort of 79 patients, we aimed to formulate a simpler prescription equation. To this end, we correlated the ratio of IA to the renal absorbed dose estimated from the first cycle (GBq/Gy, obtained by the 3TP or the 2TP methods) with the patient’s weight, lean body weight (LBW), BSA, eGFR, and the products of eGFR with each of the three body size descriptors. Then, for each of these seven correlations, we performed a linear regression forced through the origin (eliminating the intercept) and calculated the relative errors of the predicted renal GBq/Gy using the slope of the linear regression.

We also compared the accuracy of predicting the renal GBq/Gy in any given non-initial cycle with that from the previous cycle or with the average renal GBq/Gy of the two previous cycles, as we have initially been doing in our P-PRRT trial [6].

### Statistical methods

Data are presented as median and interdecile range or as mean ± SD according to the data distribution using D’Agostino-Pearson omnibus normality test. Ranges are also reported. Pearson or Spearman correlations were used depending on the normality of the data. A difference was considered as statistically significant if the *P* value was below 0.05. Correlations and linear regressions were performed using GraphPad Prism software (version 7, GraphPad Software Inc., La Jolla, CA, USA).

## Results

### Accuracy of simplified dosimetry methods

Tissue-specific effective half-lives derived from monoexponential fitting of the activity concentrations measured on days 1 and 3 are presented in Table 2. The per-cycle dosimetry results obtained with the 3TP and the 2TP methods are summarized in Table 3. For the kidney, there was only one patient case during which no biological elimination of activity between days 1 and 3 was observed. There were 30 such cases for the BM_self_ and 26 for Tumor_max_. In these cases, the 3TP_TC_ and 2TP_C_ methods were applied, while 3TP_TM_ and 2TP_M_ methods were used for all other cases. The 3TP and 2TP data (i.e., 3TP_TM_ pooled with 3TP_TC_, and 2TP_M_ pooled with 2TP_C_) were very highly correlated (Spearman *r* > 0.99, *P* < 0.0001 for all tissues). The median relative errors between the methods were small, particularly for the kidney and the tumor (≤ 2%).

**Table 2:** Tissue-specific effective half-lives derived from activity concentration at day 1 and day 3, and absorbed doses per injected activity for the 3TP reference method (*n* = 279)

	Effective half-life (h)^a^	Absorbed dose per injected activity (Gy/GBq)
Kidney	46.6 [36.3–55.7] (24.3–161.0)	0.54 [0.31–0.88] (0.21–4.25)
Bone marrow_self_	72.3 [44.9–161.0] (29.4–161.0)	0.031 [0.014–0.087] (0.004–0.258)
Bone marrow_cross_^b^	66.9 [50.3–91.6] (24.6–121.6)	0.0030 [0.0016–0.0059] (0.0005–0.0161)
Bone marrow_total_	–	0.035 [0.018–0.092] (0.009–0.262)
Tumor_max_^c^	100.9 [60.0–158.4] (27.7–161.0)	3.8 [1.0–8.6] (0.1–32.0)

Data is presented as median [interdecile range] (range)

^a^In cases of biological accumulation of activity (kidney, *n* = 1; bone marrow_self,_ *n* = 30; tumor_max_, *n* = 26), effective half-life was assumed to be equal to the physical half-life of ^177^Lu, i.e., 161 h

^b^Bone marrow cross-dose is derived from the gamma contribution of whole-body activity retention over time

^c^*n* = 278

**Table 3:** Per-cycle dosimetry estimates obtained with three-, two-, and one-time-point methods (*n* = 279)

	Absorbed dose (Gy)							Correlation vs. 3TP (*r*)^b^		Relative error vs. 3TP (%)	
	Biological decay		No biological decay^a^		Combined						
	3TP_TM_	2TP_M_	3TP_TC_	2TP_C_	3TP	2TP	1TP_D3_	2TP	1TP_D3_	2TP	1TP_D3_
Kidney	4.6 [2.7–6.6] (1.6–10.5)	4.7 [2.8–6.8] (1.6–11.1)	2.5	2.6	4.6 [2.7–6.6] (1.6–10.5)	4.7 [2.7–6.8] (1.6–11.1)	4.8 [2.7–7.1] (1.4–11.6)	0.997	0.990	2.0 [− 0.6–4.9] (− 3.4–16.0)	5.8 [− 0.4–9.2] (− 37.7–17.0)
Bone marrow_self_	0.26 [0.12–0.63] (0.05–2.08)	0.25 [0.11–0.62] (0.04–2.18)	0.36 [0.21–0.90] (0.17–3.84)	0.34 [0.20–0.86] (0.16–3.88)	0.27 [0.12–0.64] (0.05–3.84)	0.25 [0.12–0.63] (0.04–3.88)	0.22 [0.11–0.56] (0.04–2.41)	0.996	0.774	− 5.2 [− 11.4–0.6] (− 21.9–14.1)	−10.0 [− 35.4–1.3] (− 43.3–17.0)
Bone marrow_cross_	0.024 [0.013–0.061] (0.004–0.159)	0.022 [0.011–0.060] (0.003–0.155)	–	–	0.024 [0.013–0.061] (0.004–0.159)	0.022 [0.011–0.060] (0.003–0.155)	0.022 [0.012–0.054] (0.003–0.122)	0.998	0.990	− 5.0 [− 12.6 to − 0.8] (− 29.2–10.0)	−6.7 [− 16.5–1.3] (− 35.4–9.7)
Tumor_max_^c^	30.9 [7.5–70.8] (0.5–277.6)	31.3 [7.8–71.9] (0.5–271.1)	47.0 [12.1–81.0] (2.9–120.0)	47.6 [7.4–82.6] (2.9–121.7)	31.2 [7.4–74.1] (0.5–277.6)	31.7 [7.6–76.0] (0.5–271.1)	27.8 [6.8–61.4] (0.5–215.3)	1.000	0.651	1.7 [− 0.4–3.9] (− 3.5–14.0)	− 9.6 [− 30.4–8.0] (− 35.9–15.6)

*C* constant, *D3* day 3, *M* monoexponential, *TC* trapezoid-constant, *TM* trapezoid-monoexponential, *TP* time point(s)

Data is presented as median [interdecile range] (range)

Median injected activity was 7.7 (range, 0.7–32.4) GBq

^a^Kidney, *n* = 1; BM_self_ *n* = 30; BM_cross_ *n* = 0; Tumor_max_ *n* = 26

^b^Spearman’s correlation, *P* < 0.0001 in all cases

^c^*n* = 278

The results of applying the single-measurement method proposed by Hänscheid et al. [8] to our day 3 QSPECT uptake data (1TP_D3_) are shown in column 8 of Table 3. We obtained the same median error for the kidney as Hänscheid (6% at 72 h) with a comparable interdecile range. Thus, our dosimetry results, based on tomographic data acquisition, validate this practical approach, which was devised using planar imaging data. Further, despite the different imaging techniques, we obtained a similar median effective half-life for the kidney (47 h, Table 2; vs. 51 h in [8]), although we observed a wider inter-patient variability. For Tumor_max_, the 1TP_D3_ technique was slightly less accurate when applied to our data, but the range of errors was comparable.

**Fig. 3:**
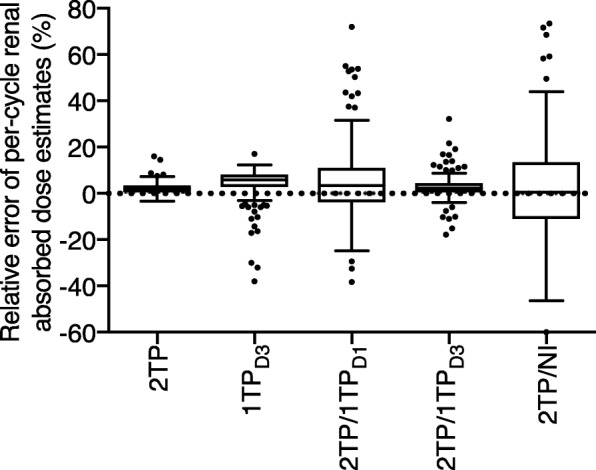
Comparison of the relative errors of per-cycle renal absorbed dose estimates obtained by the simplified methods relative to the three-time-point (3TP) method. Boxes represent the interquartile range, and whiskers the interdecile range (2TP and 1TP_D3_, *n* = 279; 2TP/1TP_D1_, 2TP/1TP_D3_, and 2TP/NI, *n* = 173)

Table 4 shows our results for the hybrid methods. In all cases, the 2TP method was applied in the first cycle. In this analysis, 2TP/1TP_D3_ was found to be more accurate than both 2TP/1TP_D1_ and 2TP/NI. The latter method yielded particularly inaccurate results for Tumor_max_, due to the interference of therapeutic response. Please note that for all tissues, we obtained median errors closer to zero with 2TP/1TP_D3_ than with 1TP_D3_ (Table 3). For the kidneys, among all the simplified dosimetry methods, 2TP was found to be the most accurate when compared to 3TP, on a per-cycle basis (Fig. 3).

**Table 4:** Per-cycle dosimetry estimates obtained with hybrid methods based on two time points for the first cycle and one time point or no imaging at all for subsequent cycles (induction cycles only, *n* = 173)

	Absorbed dose (Gy)				Correlation vs. 3TP (*r*)^a^			Relative error vs. 3TP (%)		
	3TP	2TP/1TP_D1_	2TP/1TP_D3_	2TP/NI	2TP/1TP_D1_	2TP/1TP_D3_	2TP/NI	2TP/1TP_D1_	2TP/1TP_D3_	2TP/NI
Kidney	4.8 [2.9–6.6] (1.6–8.7)	5.1 [3.0–7.1] (1.8–11.3)	4.9 [3.0–6.9] (1.7–9.2)	4.7 [2.7–7.3] (1.1–10.5)	0.887	0.985	0.807	3.4 [− 9.5–26.8] (− 38.3–86.3)	2.2 [− 2.0–7.4] (− 17.9–32.1)	0.6 [− 23.5–27.8] (− 60.2–106.3)
Bone marrow_self_	0.29 [0.14–0.65] (0.06–3.84)	0.22 [0.11–0.73] (0.06–2.33)	0.24 [0.13–0.64] (0.06–2.83)	0.24 [0.10–0.65] (0.04–3.85)	0.733	0.939	0.707	−7.0 [− 56.6–76.3] (− 73.9–155.8)	−7.2 [− 33.2–22.4] (− 44.2–51.0)	−13.8 [− 56.0–65.3] (− 93.8–370.9)
Bone marrow_cross_	0.023 [0.011–0.052] (0.005–0.100)	0.022 [0.011–0.054] (0.003–0.113)	0.021 [0.010–0.050] (0.004–0.107)	0.022 [0.010–0.060] (0.003–0.141)	0.966	0.991	0.907	−2.2 [− 21.2–14.9] (− 55.1–66.4)	−4.2 [− 15.7–2.7] (− 37.3–22.6)	1.3 [− 35.7–40.8] (− 52.5–113.0)
Tumor_max_^b^	29.7 [6.2–65.9] (1.7–120.0)	32.8 [7.0–66.6] (1.5–104.5)	31.2 [6.2–66.8] (1.9–102.1)	45.6 [9.9–113.9] (3.2–235.1)	0.946	0.980	0.713	3.6 [− 20.4–39.0] (− 46.7–141.8)	2.3 [− 11.5–21.6] (− 30.2–65.3)	31.0 [− 13.0–192.5] (− 49.9–6171.7)

*D1* day 1, *D3* day 3, *NI* no imaging, *TP* time point(s)

Data is presented as median [interdecile range] (range)

Median injected activity was 7.8 (range, 0.7–32.4) GBq

^a^Spearman’s correlation, *P* < 0.0001 in all cases

^b^*n* = 172

**Fig. 4:**
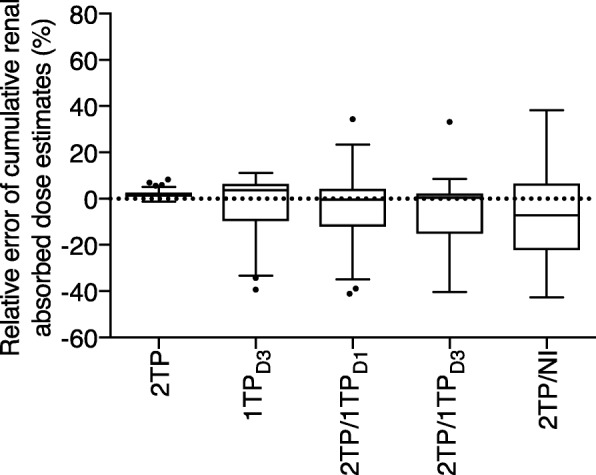
Comparison of the relative errors of per-induction course cumulative renal absorbed dose estimates obtained by the simplified methods relative to the three-time-point (3TP) method. Boxes represent the interquartile range, and whiskers the interdecile range (*n* = 65)

As the aim of the induction course of our P-PRRT regime is to deliver a given prescribed renal absorbed dose, e.g., 23 Gy in patients without significant bone marrow or renal function impairment, we compared the accuracy of the simplified dosimetry methods to that of the 3TP method, for the assessment of the cumulative dosimetry in patients having completed at least three of the four intended induction cycles (Table 5). From all the simplified approaches, the 2TP method was by far the most accurate, in particular for the kidneys (Fig. 4). Using the latter, the cumulative renal and Tumor_max_ absorbed dose for all patients agreed to within only 9% and 5%, respectively, with the corresponding absorbed doses derived from 3TP scanning (Table 5) confirming the small influence of the day 0 scan and early kinetics on the precision of dosimetry estimates. Even the total BM dose was quite accurately estimated using the 2TP protocol. When compared with 2TP, median errors increased, and error ranges widened for all 1TP-based techniques and even more so if no imaging was done on subsequent cycles.

**Table 5:** Cumulative dosimetry estimates obtained in patients having completed three or four evaluable induction cycles (*n* = 65)

	Absorbed dose (Gy)						Relative error vs. 3TP (%)				
	3TP	2TP	1TP_D3_	2TP/1TP_D1_	2TP/1TP_D3_	2TP/NI	2TP	1TP_D3_	2TP/1TP_D1_	2TP/1TP_D3_	2TP/NI
Kidney	19.3 [11.8–23.6] (6.5–26.3)	19.9 [11.8–24.3] (6.6–26.7)	18.2 [10.7–24.4] (4.5–27.2)	17.7 [11.1–24.5] (6.1–27.0)	17.4 [10.6–23.5] (4.6–26.6)	17.2 [9.9–25.3] (4.5–29.3)	1.9 [0.1–3.8] (− 1.1–8.2)	3.8 [− 24.2–6.3] (− 39.3–11.1)	− 0.5 [− 26.1–9.6] (− 41.1–34.4)	0.4 [−28.1–6.4] (− 40.4–33.2)	− 7.1 [− 32.4–18.0] (− 42.8–38.3)
Bone marrow_total_	1.10 [0.65–2.23] (0.52–9.59)	1.07 [0.57–2.15] (0.48–9.68)	0.89 [0.56–1.97] (0.38–6.9)	0.80 [0.48–2.59] (0.32–7.37)	0.86 [0.57–2.35] (0.38–8.05)	0.86 [0.50–2.35] (0.40–9.51)	−5.4 [− 9.5 to − 1.4] (− 19.0–3.9)	13.3 [− 20.5 to − 4.1] (− 33.2 to − 0.7)	− 15.7 [− 41.4–23.9] (− 61.2–68.8)	−14.4 [− 24.7–9.0] (− 57.7–66.4)	−20.3 [− 45.0–27.5] (− 72.9–110.3)
Tumor_max_	129.0 [38.7–268.2] (15.3–473.2)	131.9 [39.9–271.0] (15.8–466.2)	107.6 [34.1–205.3] (16.8–390.6)	118.8 [34.1–216.6] (15.4–402.9)	118.9 [33.6–210.3] (13.0–416.1)	155.4 [38.8–341.6] (14.0–575.2)	1.4 [− 0.2–3.3] (− 1.5–4.3)	−16.1 [− 45.9–3.2] (− 60.0–12.0)	−5.6 [− 41.0–10.3] (− 62.7–39.5)	−2.9 [− 40.2–6.4] (− 63.4–21.9)	15.9 [− 29.2–80.5] (− 75.6–400.5)

*D1* day 1, *D3* day 3, *NI* no imaging, *TP* time point(s)

Data is presented as median [interdecile range] (range)

Median cumulative injected activity was 30.5 (11.9–78.6) GBq

### Inter-observer variability

Table 6 illustrates the inter-observer variability of the per-cycle and cumulative renal, BM_total_, and Tumor_max_ absorbed doses assessed independently by the three observers in 15 patients (60 cycles) using three methods: 3TP, 2TP, and 2TP/1TP_D3_. There was an excellent inter-observer agreement between all observers for the kidney using the three methods, the best agreement being for the cumulative renal dose estimated using the 3TP and 2TP methods. The span of errors was larger for Tumor_max_, owing it to variations in the precise placement of the VOI over the most intense region of the dominant lesion. For both BM_total_ and Tumor_max_, there was a trend towards a lesser inter-observer agreement when the least experienced observer (observer 3) was involved. BM_total_ reproducibility suffered from the low-level and noisy uptake data in the BM compartment, and consequently BM_self_, the dominant component of BM_total_, was more sensitive to the position of the VOI than were the absorbed doses of the other tissues of interest. Nevertheless, the inter-observer agreement on the cumulative BM_total_ dose was fair.

**Table 6:** Inter-observer variability of dosimetry estimates in 60 induction cycles received by 15 patients

	Relative error (%)		
	Kidney	Bone marrow_total_	Tumor_max_
Observer 2 vs. observer 1			
Per cycle			
3TP	− 0.2 ± 3.4 (− 7.8–10.0)	− 2.4 ± 20.4 (− 53.7–75.9)	− 0.2 ± 8.6 (− 34.1–40.5)
2TP	− 0.3 ± 3.4 (− 9.0–8.2)	− 2.2 ± 20.9 (− 50.9–7.8)	− 0.2 ± 8.2 (− 33.0–38.4)
2TP/1TP_D3_	− 0.5 ± 3.4 (− 8.8–7.8)	− 11.2 ± 25.5 (− 98.2–30.6)	−1.9 ± 6.2 (− 33.0–6.4)
Cumulative			
3TP	− 0.2 ± 2.3 (− 2.9–3.9)	− 4.5 ± 8.9 (− 24.0–7.8)	− 0.6 ± 4.0 (− 8.3–8.0)
2TP	− 0.3 ± 2.3 (− 4.2–3.6)	− 4.4 ± 8.8 (− 21.5–8.0)	− 0.6 ± 3.8 (− 7.8–7.1)
2TP/1TP_D3_	− 0.5 ± 2.3 (− 3.9–4.0)	− 12.1 ± 19.7 (− 68.5–11.4)	− 6.1 ± 10.1 (− 32.2–4.5)
Observer 3 vs. observer 1			
Per cycle			
3TP	− 0.5 ± 4.7 (− 21.5–9.4)	3.6 ± 29.0 (− 31.2–153.2)	− 4.6 ± 9.0 (− 42.2–14.2)
2TP	− 0.3 ± 4.5 (− 21.4–8.2)	4.2 ± 29.9 (− 33.5–159.3)	− 4.3 ± 8.7 (− 39.9–13.5)
2TP/1TP_D3_	− 0.7 ± 5.8 (− 35.7–9.4)	− 16.3 ± 35.9 (− 98.0–57.9)	− 2.9 ± 8.6 (− 39.9–15.0)
Cumulative			
3TP	− 0.4 ± 1.5 (− 2.6–3.5)	1.9 ± 12.0 (− 13.2–31.4)	− 3.9 ± 5.2 (− 11.0–10.7)
2TP	− 0.2 ± 1.2 (− 1.6–3.3)	2.3 ± 12.3 (− 13.7–31.9)	− 3.7 ± 5.0 (− 10.7–10.1)
2TP/1TP_D3_	− 0.6 ± 2.8 (− 9.5–3.9)	− 17.3 ± 25.8 (− 74.7–3.3)	− 5.5 ± 11.0 (− 33.8–13.5)
Observer 3 vs. observer 2			
Per cycle			
3TP	− 0.2 ± 5.1 (− 24.0–13.1)	7.3 ± 23.2 (− 30.0–82.2)	− 4.1 ± 9.4 (− 28.4–25.2)
2TP	0.0 ± 4.8 (− 22.6–10.0)	7.8 ± 23.9 (− 29.8–92.5)	− 3.8 ± 9.1 (− 27.5–23.6)
2TP/1TP_D3_	− 0.2 ± 6.4 (− 38.4–10.3)	− 3.7 ± 33.9 (− 93.0–92.5)	− 0.9 ± 7.4 (− 14.8–24.0)
Cumulative			
3TP	− 0.2 ± 2.0 (− 3.6–3.0)	7.0 ± 11.4 (− 7.9–34.3)	− 3.2 ± 7.8 (− 17.5–20.7)
2TP	0.1 ± 2.2 (− 3.9–3.6)	7.2 ± 11.0 (− 8.6–29.2)	− 2.9 ± 7.4 (− 16.6–19.4)
2TP/1TP_D3_	− 0.1 ± 3.4 (− 10.3–3.3)	− 4.4 ± 25.6 (− 68.2–27.9)	1.0 ± 10.4 (− 8.0–27.8)

*D3* day 3, *TP* time point(s)`

Data is presented as mean ± SD (range)

### Accuracy of activity prescription at first and subsequent cycles

**Fig. 5:**
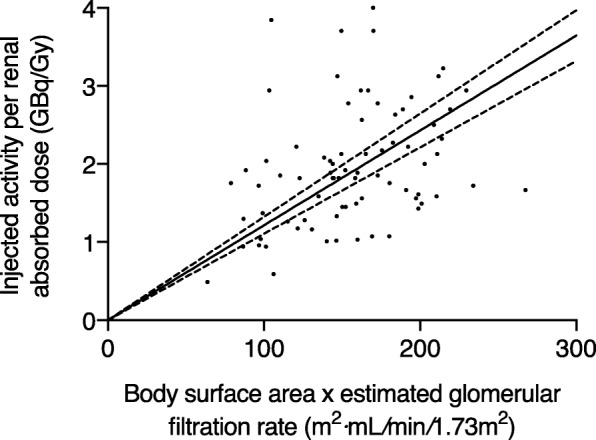
Injected activity per renal absorbed dose at the first cycle vs. the product of body surface area and estimated glomerular filtration rate (*n* = 77). There was a moderate correlation between variables (Spearman *r* = 0.39, *P* = 0.0005). The slope of the linear regression curve forced through origin (solid line; 95% confidence interval, dashed lines), which was 0.012 GBq · min *×* 1.73/mL*/*Gy, is to be used to adjust the injected activity at the first cycle in a personalized PRRT protocol

**Fig. 6:**
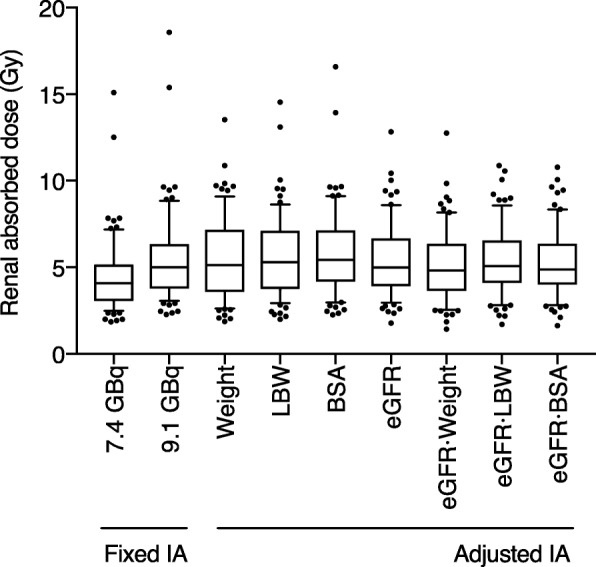
Comparison of renal absorbed dose delivered during the first cycle of fixed injected activity (IA) vs. personalized PRRT regimes (*n* = 77). In the latter, the prescribed renal absorbed dose is 5 Gy and the IA is adjusted based on weight, lean body weight (LBW), body surface area (BSA), estimated glomerular filtration rate (eGFR), or the product of eGFR and of a body size descriptor. For comparison, a fixed IA of 9.1 GBq would yield a median renal absorbed renal absorbed dose of 5 Gy

Correlations and linear regression slopes between the body size descriptors, the eGFR, or their products vs. the IA per renal absorbed dose at the first induction cycle are reported in Table 7. The strongest correlations were found when using either eGFR · LBW or eGFR · BSA (Fig. 5) as predictors of renal GBq/Gy, and both seem equally appropriate for personalized IA prescription at the first cycle (Fig. 6). We therefore elected to continue using eGFR and BSA for determining IA at the first cycle and updated our initial formula (found in [6]) with this simpler equation:

1 $$ \mathrm{Personalized}\ \mathrm{AI}\ \left(\mathrm{GBq}\right)=K\cdotp \mathrm{eGFR}\ \left(\mathrm{mL}/\min /1.73{\mathrm{m}}^2\right)\cdotp \mathrm{BSA}\ \left({\mathrm{m}}^2\right)\cdotp \mathrm{Prescribed}\ \mathrm{renal}\ \mathrm{absorbed}\ \mathrm{dose}\ \left(\mathrm{Gy}\right) $$

**Table 7:** Correlation between body size predictors, eGFR, and the IA per renal absorbed dose (GBq/Gy) at the first induction cycle (*n* = 77)

	Correlation ^a^				Linear regression slope		Relative error (%)	
	3TP		2TP		3TP	2TP	3TP	2TP
	*r*	*P*	*r*	*P*				
Weight (Kg)	0.13	0.25	0.13	0.26	0.026	0.026	2.3 [−46.0–66.6] (− 61.5–165.7)	2.0 [−47.0–66.3] (− 62.6–168.8)
LBW (Kg)	0.18	0.12	0.17	0.13	0.037	0.036	5.6 [−38.4–73.9] (− 58.4–190.4)	6.8 [− 39.4–71.0] (− 59.6–193.7)
BSA (m^2^)	0.16	0.17	0.15	0.13	1.09	1.07	7.4 [−38.2–73.5] (− 53.5–227.7)	6.9 [− 39.1–76.8] (− 54.8–231.5)
eGFR (ml/min/1.73m^2^)	0.34	0.002	0.36	0.001	0.022	0.021	1.0 [− 36.2–70.2] (− 61.2–164.1)	1.5 [− 36.2–71.9] (− 61.1–173.3)
eGFR · weight	0.34	0.002	0.35	0.002	0.00026	0.00026	−1.9 [− 46.7–63.8] (− 62.9–124.4)	−2.7 [− 45.9–61.7] (− 62.8–123.3)
eGFR · LBW	0.40	0.0003	0.41	0.0002	0.00037	0.00036	−0.3 [− 43.0–57.1] (− 67.6–140.2)	0.8 [− 43.4–58.3] (− 67.5–139.0)
eGFR · BSA	0.38	0.0008	0.39	0.0005	0.012	0.012	− 0.0 [−36.2–70.2] (− 63.8–128.2)	− 0.7 [− 36.9–70.1] (− 63.7–127.3)

*BSA* body surface area, *eGFR* estimated glomerular filtration rate, *LBW* lean body weight, *TP* time points

Relative error data is presented as median [interdecile range] (range)

^a^Spearman’s correlation

where *K* = 0.012 GBq · min · 1.73/mL*/*Gy, i.e., the slope of the linear regression (Fig. 5).

For the subsequent cycles, the median error of the renal GBq/Gy relative to that of the previous cycle was − 0.7% (interdecile range, − 21.0 to 20.2%; range, − 49.1 to 54.0%; *n* = 194) for the 3TP method, − 0.8% (interdecile range, − 20.4 to 19.0%; range, − 49.4 to 50.2%; *n* = 194) for the 2TP method, and − 0.3% (interdecile range, − 20.5 to 18.6%; range, − 40.2 to 87.4%; *n* = 168) for the 2TP/1TP_D3_ method. When in the analysis of the third and fourth induction cycle the average of the renal GBq/Gy of the two prior cycles was used, the resulting errors were − 0.9% (interdecile range, − 21.3–19.1%; range, − 49.9–54.4%; *n* = 192), − 0.6% (interdecile range, − 21.4–18.6%; range, − 48.5–54.5%; *n* = 192) and − 0.6% (interdecile range, − 19.8–19.6%; range, − 45.7–75.3%; *n* = 166), respectively. Hence, unlike in our initial P-PRRT protocol, these results convinced us that averaging the renal GBq/Gy from the two prior cycles (instead of just one) does not significantly improve the precision of the IA prescription. The IA prescription for the subsequent cycle has been updated in our P-PRRT protocol as follows:

2 $$ \mathrm{Personalized}\ \mathrm{IA}\ \left(\mathrm{GBq}\right)=\mathrm{Prior}\ \mathrm{cycle}\ \mathrm{IA}\ \mathrm{per}\ \mathrm{renal}\ \mathrm{dose}\ \left(\mathrm{GBq}/\mathrm{Gy}\right)\cdotp \mathrm{Prescribed}\ \mathrm{renal}\ \mathrm{dose}\ \left(\mathrm{Gy}\right) $$

## Discussion

The widely adopted one-size-fits-all PRRT protocol, i.e., four induction cycles of 7.4 GBq ^177^Lu-octreotate, has been initially devised in 2001 based on the dosimetry data from only five patients [3]. Since that time, dosimetry has not been routinely performed in most centers (including for PRRT administered in the NETTER-1 trial [2]). This fixed IA regime is known to yield highly variable absorbed doses to critical organs, but because these fall well below conservative safety thresholds (e.g., 23 Gy for the kidney) in the vast majority of patients, this confers to PRRT a very favorable safety profile [1, 2]. In parallel, current cure rates are marginal, suggesting that most patients are being undertreated with the empiric PRRT regime [1, 2].

Escalating tumor absorbed dose could potentially improve the efficacy of PRRT, although realistically, this cannot be done through a conventional empiric IA escalation without compromising the excellent safety record of PRRT. To optimize tumor irradiation at the patient level, we and others have proposed to optimize the renal absorbed dose by either personalizing the IA per cycle or the number of induction cycles [4, 6]. These two approaches resulted in increased cumulative IA and tumor absorbed dose in the majority of patients [4, 6]. Further, when administering personalized IA, our preliminary results suggest a similar short-term side effect and toxicity profile as those observed when using the empiric PRRT regime [10]. Although our outcome data is not yet mature enough to document significantly improved clinical outcomes, nevertheless, we believe that personalized radionuclide therapy is more faithful to the principles of radiation oncology, where the absorbed doses are prescribed and monitored. In internal radiotherapy, this could be done through IA personalization and routine dosimetry.

Despite the fact that our imaging protocol did not include a late time point, we obtained similar median renal absorbed doses per IA (median, 0.54 Gy/GBq) to those observed by Sandstrom et al. (medians, 0.62 and 0.59 Gy/GBq for the right and the left kidneys, respectively), who also used QSPECT and small-VOI sampling, but scanned patients until day 7 [5]. The concordance between our results is consistent with the fact that the renal activity concentration decays moxoexponentially after 24 h, as demonstrated by Handshied et al. [8].

The median BM absorbed dose we obtained when using our QSPECT-based method (0.035 Gy/GBq) is well within the range of estimates published by others using various techniques based on imaging, blood, and urine sampling [5, 11]. Furthermore, this result is particularly close to the mean BM absorbed dose reported by Svensson et al. (0.027 Gy/GBq), which was derived from imaging data only and included a later time point [12, 13]. The correlation between QSPECT-based BM absorbed dose estimates and subacute thrombocytopenia provides initial clinical validation of our technique [6]. However, in patients with bone metastases, the BM dosimetry estimates may be less reliable, as even if obvious bone metastases were avoided when placing the BM VOIs, we cannot rule out the influence of non-apparent micrometastases or diffuse BM infiltration.

Dosimetry is an essential component of P-PRRT but is often perceived by the medical community as being too complex, or by the physics community as not accurate enough. However, SPECT/CT cameras are now widely available, and simple ^177^Lu calibration methods have been proposed [7, 14], facilitating implementation of individualized dosimetry based on QSPECT in the clinics. Performing dosimetry calculation based on simplified activity concentration sampling methods, such as the small-VOI method used in this study, is more practical to perform than the full organ segmentation while yielding similar results and is more accurate than planar imaging-based dosimetry [15]. For these reasons, we have routinely been performing dosimetry using a 3TP QSPECT scanning schedule along with the small-VOI sampling. But still, many would see dosimetry as resource-consuming. This opinion would be based on the general beliefs that a minimum of three measurements are necessary [16] or that the scanning protocol must include late time points, such as up to 4 to 7 days [17]. Such requirements tend to increase both the clinical burden and the patient inconvenience when performing dosimetry, and as such constitute barriers to its wide clinical adoption. Conversely, simplified dosimetry approaches having a clinically relevant level of accuracy could facilitate making dosimetry a standard of care, not just for monitoring purposes, but also for personalizing radionuclide therapy. To overcome the issues discussed above, the primary objective of this study was to further simplify our dosimetry methods.

The 2TP method offers an excellent accuracy for both the per-cycle and the cumulative absorbed dose estimates relative to the 3TP method, in particular for the kidney and the tumor, which convinced us to abandon the day 0 scan. Further, our results validated the 1TP_D3_ technique proposed by Hänscheid and co-workers [8]. While they advocate scanning on day 4 to achieve the best accuracy for both the kidney and the tumor, scanning on day 3 is considered more practical in our setting, offers about the same accuracy for the kidney dosimetry and a very reasonable accuracy for the tumor. This 1TP_D3_ method is an appealing alternative to 2TP, although accuracy could be slightly improved, at least for the kidney and Tumor_max_, by simply adding a second imaging time point during the first cycle and then applying the effective decay constant to the one-time samples during subsequent cycles (2TP/1TP). This hybrid method is more accurate when, for the non-initial cycles, the imaging is performed on day 3 (2TP/1TP_D3_) rather than on day 1 (2TP/1TP_D1_). This is likely because, in individual patients, day 3 measurements are better correlated with the integrated TACs (i.e., absorbed doses) than are those performed on day 1 and, as such, are less sensitive to small intra-patient cycle-to-cycle differences in tissue uptake and kinetics [8]. However, since in parallel we alter the IA prescription based on renal dosimetry, we prefer pursuing our P-PRRT program with the 2TP protocol, which offers, in our opinion, the best balance between high accuracy and practicality. Importantly, we would not recommend not imaging patients at subsequent cycles and extrapolating dosimetry from the first cycle (2TP/NI), assuming constant Gy/GBq in tissues (i.e., completely ignoring any cycle-to-cycle difference in uptake or kinetics), as this approach causes the uncertainty of the resulting absorbed dose estimates to increase, in particular for tumor, which can be affected by the therapeutic response. The clinical burden of the 2TP schedule in terms of the camera and personnel time is reasonable and comparable to that of performing an ^111^In-octreotide scan. Furthermore, performing the last scan on the third day limits the inconvenience for the out-of-city patients.

A very good inter-observer agreement has already been reported for renal dosimetry, with median errors of less than 5% for the small-VOI dosimetry method [17]. Our results confirm these observations. Of note, we intentionally chose observers with different backgrounds and have shown that even dosimetry estimates from our novice observer (first-year medical student) were well in agreement with those from more experienced observers, suggesting that a reasonably reproducible activity concentration sampling technique is easily attainable with a relatively short training. Also, the whole processing of one patient case, including VOI drawing and data transfer to the spreadsheet or database, can be performed in about 15 to 20 min. We are contemplating to train nuclear medicine technologists to perform PRRT dosimetry under medical supervision, eventually making them sub-specialized as nuclear dosimetrists.

Finally, we revisited our personalized IA prescription scheme for our P-PRRT protocol. For the first cycle, we derived a simpler equation to determine the personalized IA than the one we initially suggested [6]. The latter is still based on the product of eGFR and BSA, but eGFR · LBW would have provided a similar level of predictive accuracy. We acknowledge that this accuracy is at most moderate and comparable to that of an initial fixed IA in terms of interquartile or interdecile range (Fig. 6). However, the main advantage of personalizing the first cycle IA is to avoid extreme cases of overdosing, such as delivery 18 Gy to the kidney when administering a fixed IA of 9.1 GBq to every patient (Fig. 6). Rather, personalizing IA could limit the renal absorbed dose to 11 Gy, for the same median of 5 Gy. When ^68^Ga-octreotate PET will be routinely performed in all our PRRT patients, we will explore adding the pre-treatment tumor sink effect analysis into the prescription scheme, which could potentially improve the predictive accuracy of the model [18].

## Conclusions

We propose a ^177^Lu dosimetry protocol based on two-time-point QSPECT imaging and the small-VOI sampling, which yields accurate dosimetry results, particularly for the kidney and the tumor, with a very high inter-observer reproducibility. Performing the last QSPECT/CT scan no later than on the third day post-PRRT increases patient convenience, particularly for the out-of-city patients who travel to receive PRRT. Pragmatic ^177^Lu dosimetry methods could facilitate the practice of personalized radionuclide therapies, including the rapidly emerging prostate-specific membrane antigen radioligand therapy.

## Abbreviations

ACDF: Activity concentration dose factor
BSA: Body surface area
CT: Computed tomography
eGFR: Estimated glomerular filtration rate
IA: Injected activity
LBW: Lean body weight
NET(s): Neuroendocrine tumor(s)
NI: No imaging
PET: Positron emission tomography
P-PRRT: Personalized PRRT
PRRT: Peptide receptor radionuclide therapy
QSPECT: Quantitative SPECT
SPECT: Single-photon emission computed tomography
SUV: Standardized uptake value
TAC(s): Time-activity curve(s)
TP: Time point
VOI(s): Volume(s) of interest
